# The Story of the Insane from Year to Year

**Published:** 1899-05-06

**Authors:** 


### THE STORY OF THE INSANE FROM YEAR TO YEAR.

(Eleventh Series.)

### IPSWICH BOROUGH ASYLUM.

On the first day of 1897 there were 265 patients in this asylum, and by the close of the year the numbers had risen to 285. There were 82 first admissions, 9 readmissions, 22 recoveries, and 34 deaths. The percentage of recoveries, calculated on the admissions, stands at 24·2, which is lower than their average by 7 per cent. Indeed, we notice that on the male side the percentage of recoveries is only 16·6, while on the women's side it is 40 per cent. The conditions of these departments must be practically identical; yet the recovery rate varies extremely, and shows that such variation is due, chiefly, to the class of patients admitted during the year and during the previous year. Fourteen of the deaths are accounted for by cerebral and spinal diseases, and 4 by consumption. The total number of deaths yields a percentage of 11·93 on the average number resident. This is rather higher than the average of public asylums, and here again it is interesting to point out that on the male side the death rate was 19 per cent., and only 7 per cent. on the female side. Various moral causes account for 11 of the admissions, intemperance in drink for 13, hereditary tendency for 18, and previous attacks for 20. There is not much of special interest in any of the reports accompanying the statistical tables. The weekly cost of maintenance was 10s. 1½d. per head.

KENT COUNTY ASYLUM AT MAIDSTONE.

This asylum issued its fifty-first annual report on December 31st, 1897, and it then contained 1,624 patients, the numbers having fallen from 1,663. This decrease was owing to the transference of patients to the asylum at Chartham Down. During the year 1897 there were 350 admissions, 38 readmissions, 142 recoveries, 23 discharged relieved, and 236 deaths. The recoveries stand at 37·4 per cent. of the admissions, and the deaths at 14·3 on the average number resident. Of the deaths, 72 were caused by cerebral and spinal diseases; 62 by consumption; 15 by pneumonia; 12 by enteritis; and 9 by enteric fever. Of the admissions, 29 owed their insanity to domestic troubles and other moral causes; 27 to intemperance in drink; 69 to hereditary influence; and 67 to previous attacks. The death-rate is nearly 4 per cent. above the average of the Maidstone Asylum—and as Dr. Davies points out it is only partly explained by the deaths from enteric fever and enteritis. No doubt the senile cases sent in from the unions contribute their quota, and the over-crowding helps to swell the list. In fact we have already stated that 62 of the deaths were from consumption, which, compared to other asylums, is a high proportion. It is well known that the epidemic of enteric fever which broke out in Maidstone soon spread to the asylum, and altogether 88 patients and 19 of the staff were stricken. There were 11 deaths from this disease. About the same time enteritis made its appearance, and there were 12 deaths from it. In other asylums it has been noted that while enteric fever attacks both patients and attendants in similar proportions, enteritis often spares the staff. It would be interesting to know what Dr. Davies' experience was on this particular point. The medical officers certainly deserve praise for the manner in which they coped with these epidemics. It is also satisfactory to note that neither mechanical restraint nor seclusion was employed during the year, and that there was no case of suicide. The committee say that prompt measures were taken by Dr. Jackson to stay the epidemic of fever. Dr. Davies was absent on long leave. The weekly cost of maintenance is 9s. 2½d. per head.

EASTERN COUNTIES' ASYLUM FOR IDIOTS AT COLCHESTER.

There were 251 patients in this asylum at the end of 1897. The admissions during the year were 32, the discharges 13, and the deaths 8. The usual tables of the Medico-Psychological Association are not given in this report, and, indeed, are hardly necessary in an asylum which admits idiots only. Mr. Turner, the lay superintendent, most truly says that idiocy is a terrible affliction in the homes of the poor, and he states that he recently visited "a family in which out of ten children there were three imbeciles," and he adds that the asylum contains two brothers from Suffolk and two from Essex, "and in each case there are two others at home who are equally afflicted. Four applicants on the present election list have two or more imbecile brothers or sisters; and one can perhaps imagine better than it can be described, the care and attention that these children need, and how dreadfully handicapped a mother must be under such sad circumstances." True enough; but can nothing be done to check the frightful immorality of bringing such children into the world? The asylum is spending nearly £9,000 a year, and it is receiving from all sources a sum under £8,500 a year, so that there is a considerable deficit, which, it is to be hoped, a few rich and charitable people will wipe off and leave the institution free of debt to continue its good work. Not that we altogether approve of the principle underlying this and similar establishments, because we think that the duty of providing accommodation for all classes of the insane should be imperative on the State. Already several county asylums have provided annexes for the treatment of idiot children, and we await with hope the day when this provision will be universal. In the meantime, however, we commend the Colchester Asylum for Idiots to those people who can spare their guineas; as undoubtedly good work is being done in it and a want supplied.

